# Quantifying the usage of small public spaces using deep convolutional neural network

**DOI:** 10.1371/journal.pone.0239390

**Published:** 2020-10-02

**Authors:** Jingxuan Hou, Long Chen, Enjia Zhang, Haifeng Jia, Ying Long

**Affiliations:** 1 School of Architecture, Tsinghua University, Beijing, China; 2 Department of Environmental Planning and Management, Tsinghua University, Beijing, China; 3 School of Architecture and Hang Lung Center for Real Estate, Tsinghua University, Beijing, China; 4 Key Laboratory of Eco Planning & Green Building, Ministry of Education, Tsinghua University, Beijing, China; University of Wisconsin Madison, UNITED STATES

## Abstract

Small public spaces are the key built environment elements that provide venues for various of activities. However, existing measurements or approaches could not efficiently and effectively quantify how small public spaces are being used. In this paper, we utilized a deep convolutional neural network to quantify the usage of small public spaces through recorded videos as a reliable and robust method to bridge the literature gap. To start with, we deployed photographic devices to record videos that cover the minimum enclosing square of a small public space for a certain period of time, then utilized a deep convolutional neural network to detect people in these videos and converted their location from image-based position to real-world projected coordinates. To validate the accuracy and robustness of the method, we experimented our approach in a residential community in Beijing, and our results confirmed that the usage of small public spaces could be measured and quantified effectively and efficiently.

## Introduction

Small public spaces, such as plaza, pocket parks and public spaces in residential communities, as key built environment elements that provide venues for a variety of activities [1–3], may have a major impact on the quality of people’s life and could even potentially contribute to both the physical and mental health of local residents [4]. Since 1960, a growing group of scholars have realized the significance of small public spaces, and studied on the method to explore the usage of small public spaces. Surveys and interviews are common methods to assess people's satisfaction or impression of a space [5–7]. The systematic observation [8] proposed by Barker [9] is also a classic way to record what people do and how they behave in a particular space. Inspired by Barker and his observation method, Winkel and Sasanoff [10] and Whyte [11] used grids to record people’s trajectory in public spaces. Besides, comparative experiments and workshops from psychology are also important methods for studying the relationship between environment and people’s behaviors [12]. However, most of conventional studies are based on field survey and observation on people’s behaviors, but lack the measurement of the physical environment in the space.

Recently, advances in the Information and Communication Technologies (ICTs) and sensors like internet of things (IoTs) and wearable devices are providing a new context for gaining insights into quantifying the interactions between people and physical environment. Mobile signaling data, GPS data, and LBS data become readily accessible data to facilitate the study of social activities in a meso or micro scale [13–15]. Embedded sensors, especially wearable devices make it feasible to measure and visualize people’s activities, motions and preference [16–19]. Besides, physical environment captured by Remote Sensing Imagery [20] and Street View Pictures [21–23] can be quantified now thanks to the achievement in deep learning algorithms for image processing. Table 1 lists a comprehensive literature review regarding the methods used in quantifying the usage of small public spaces. However, mobile phone data and GPS data is unable to depict the activities within 100 square meters due to their positioning accuracy, sensor-based human behavior research is limited to experimenters or a certain range at a high cost, while image-based environmental assessment and pedestrian detection are weak in reflecting the dynamic usage of small public space. With the development of deep convolutional neural network, using video-based surveillance and monitoring applications to record people’s behavior and human facial information [24], count people across some area [25] or to track someone’s route [26] provides a novel approach to observe the usage of small public spaces. Nevertheless, these initial explorations focused more on people’s behavior or activities, ignoring the interaction between people and the physical environment. In addition, the usage of small public space from the perspective of built environment is not considered in these studies.

**Table 1:** Classification of methods for measuring the usage of public space.

Category	Research Methods and Tools	Analytical Unit	Data	Spatial Granularity
Subjective judgments	Cognitive Map: mapping the elements and noting any special successes or difficulties in the potential image structure [5].	City/ Zone	Image maps	—
	Post-occupancy evaluations (POE): questionnaires for quantitative study on environmental attitudes and mobility [6, 7].	City/ Zone/ Building	Questionnaires and interview feedback	—
Systematic observation	Site survey and observation: investigating on public life and public space (PLPS) based on the pedestrian volume, and activities perceived by observers [8].	A plaza or a park	Subjective perception	—
	Grid record: calculating the number of persons in the demarcated grid in the space [11].	Grid	Videos/ Photos	2m x 2m
Data driven analysis	New data analysis and visualization: calculating the density of users [13–15].	Administration unit/ 1 km by 1 km grid/ Street blocks	Mobile signaling data/ GPS data/ LBS data/	200m x 200m/ 10m x 10m/ 10m x 10m
	Experiments with embedded sensors: using differential Wi-Fi trilateration to assess indoor position [19].	Layer/ Room	Wi-Fi data	30m x 30m
	Using Satellite Imagery to identify the land use patterns [20].	Street blocks	Satellite Imagery	30m x 30m

To fill those gaps identified, we proposed a novel approach that combines both conventional method and emerging technologies to quantify how small public spaces are being used. This is our attempt to quantify the usage of small public spaces and we simply define the usage as the footsteps of people who enters the spaces, does all types of activities, including but not limited to walking through, resting, chatting, etc. Based on Whyte’s grid analysis method [11], we embraced the technique of deep convolutional neural network and applied the perspective principle into spatial analysis, and finally achieved the goal of quantifying the usage of small public spaces effectively and efficiently. To start with, we deployed photographic devices to record videos that cover the entire public space, and then processed these videos into images. Subsequently, we adopted a deep convolutional neural network to detect the objects (human beings in this case) in these images, and geo-located people to real-world projected coordinates to generate cumulative trajectories. To validate the accuracy and robustness of our approach, we experimented it in a real-world community in Beijing.

The rest of this paper is organized as follows. Section 2 describes the methodology, including the research framework, data collecting, data preparation, object detection and geo-locating algorithm. Several validation experiments are conducted in Section 3 to examine the accuracy and applicability of our approach. The approach’s features, contributions, limitations, and suggestions for further research are discussed in Section 4.

## Methodology

**Fig 1:**
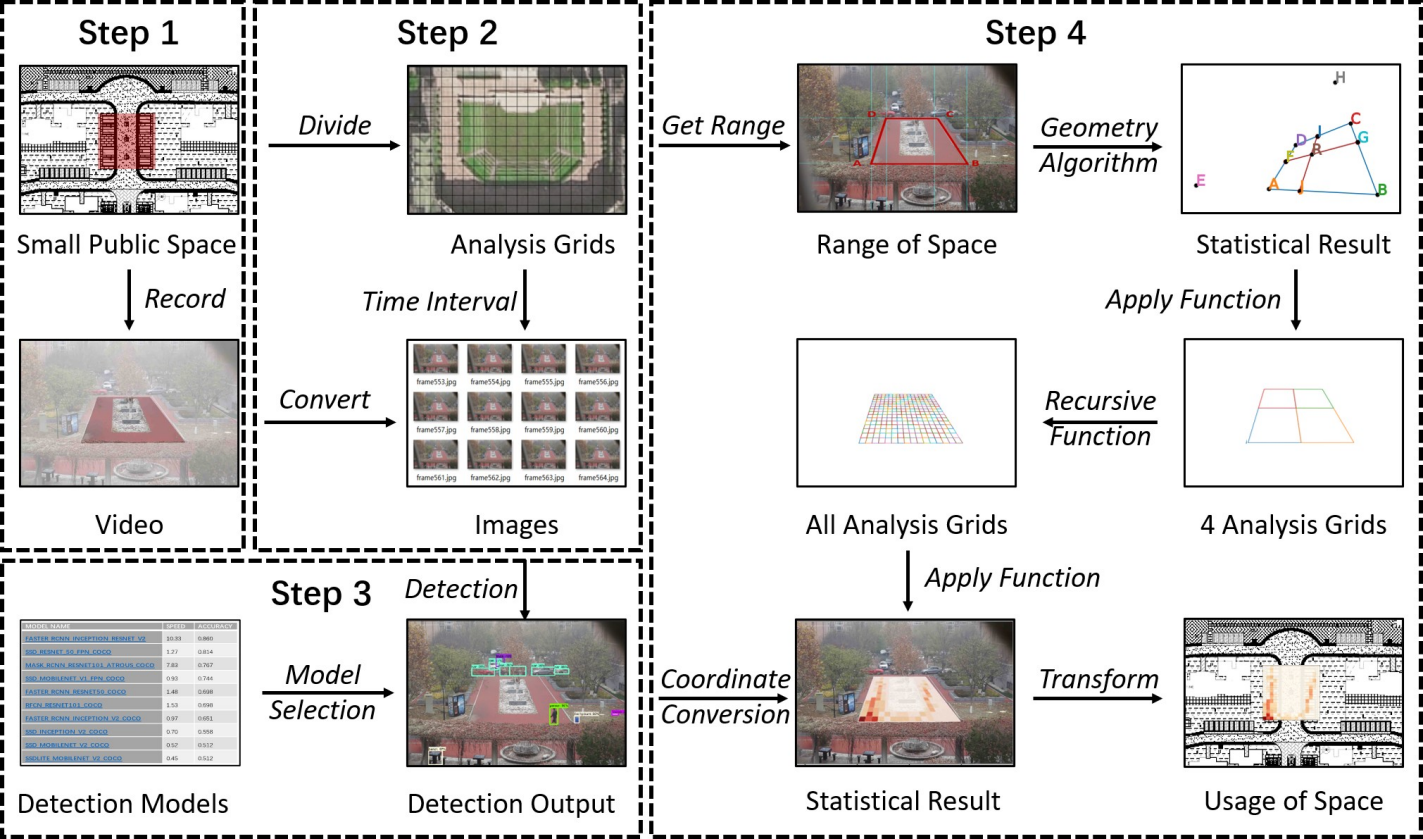
Technology roadmap of the approach.

Inspired by the grid analysis method to map the usage of public spaces proposed by Whyte [11], we built a four-step approach to achieve the similar goal in an automatic manner (Fig 1). First, videos of a small public space are recorded by photographic devices; second, videos are converted into images based on the pre-determined time interval; third, persons in these images are detected using deep convolutional neural network, and their specific locations in the images are recorded; fourth, those detected persons are geo-located from locations in images to real-world locations by using geometry algorithm and recursive function, then the final results are visualized and analyzed to illustrate the usage of the small public space. The following paragraphs demonstrate this methodology with more details.

### Data collecting

Before collecting the data, we first obtained the boundary of the study area and its minimum enclosing square by remote sensing image with coordinates in a projected coordinate system. The primary data used in this approach are videos recording the minimum enclosing square of the entire study area (small public spaces) for a period of time without severe perspective distortion. As a result, in the data collecting process, the selection and installation of photographic devices should be specified, so that these recorded videos are qualified for the following quantitative analysis. Firstly, according to the specific need of a study, the video device should be selected among those allowing extra battery extender for shooting the study area for a long period of time. Besides, the video format that a photographic device could generate would not influence the device selection.

The installation of the photographic device also matters since it determines the lens coverage and the angle of videos. According to the size of a study area, the installation of the photographic device can be classified into "high position" and "low position" based on the height of the mounted device. The "high position" means that the device is mounted higher from the ground, usually shooting from a building roof or higher-level windows. The photographic device could be installed at a “high position” if there are no obstacles, such as tree leaves and power cables that may block the view of the study area. The "low position" refers to placing the device on the top of the fence wall or on the roof of a flat house. The photographic device could be installed at a “low position” when the traffic volume in the study area is relatively low. When the traffic volume gets higher in an area covered by low position device, people located at the focal distance of the lens will blur other objects in the image due to perspective principles. Table 2 lists the detailed information of two installation type of photographic devices. After the device is mounted, it is set to automatically record videos of the study area for a certain period of time.

**Table 2:** Installation type of photographic devices.

Installation	Sample	Height	Position	Disadvantages
High Position	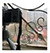	5~8m	From building roofs or high-level windows	Tree leaves and power cables that may block the view of the study area
Low Position	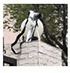	1.5~ 5m	On top of the fence wall or a flat house	When the traffic flow gets larger, people located at the focal distance of the lens will blur other objects

### Data preparation

In the data preparation process, the recorded video must be converted and filtered into images by setting a time interval according to the research purpose. Time interval refers to the length of time between two consecutive images, which determines the total number of images to be selected and processed, as well as the continuity of movements of people in images. The purpose of filtering images is to optimize the number of images to be analyzed in the object detection process, which helps to minimize the load of images while the results could still reflect the continuous movements of people. The concept “time interval” is calculated as the equation below:

TI=GW/AS*FN, (1)

where *GW* is the width of each analysis grid, *AS* is the average walking speed of pedestrian, and *FN* is the frame rate of the video.

The idea of dividing small public spaces into analysis grids is inspired by Whyte [8], who took the pavement bricks in a plaza as the analysis grids and counted the total number of persons who walked on each of them. In deciding the size of the grid (*GW*), a criterion is that there should be no more than one cluster of people (one person, a couple or a family group) in a single grid in each image. Naturally, the minimum distance between two strangers in a public space is 0.9 meters [27], so the width of each analysis grid should not be significantly greater than 0.9 meter, so as to avoid capturing more than one person in a single grid. Then in order to fulfill the optimization requirement of the image extraction, an individual moving at an average walking speed (*AS*) should not appear in a non-adjacent analysis grid in two continuous images. Since the average walking speed of a pedestrian is 1.2 meters per second [27], one frame of image is extracted from every 0.75 second (*GW/AS*). Considering that the frame rate (*FN*) in this research is 24 (one second of video is composited by 24 frames), the final time interval equals to 18 (GW/AS*FN), which means 1/18 frames are extracted from videos and would be used in further analysis.

### Object detection

In order to quantitatively measure how small public spaces are used, we proposed an approach to quantify the human activities by detecting the location of people in videos using deep convolutional neural network (CNN), a subset of deep learning and neural networks, which has been widely used in recent large-scale object detection and recognition systems because of its power in learning features [28]. Increasing literature on applying CNN for processing images, audios, and video, especially those proposing it for pedestrian detection [29, 30] inspire us for processing our data in this study.

We selected a total of 10 most commonly used deep convolutional neural networks by the time this research was conducted that support object detection modules to find a most appropriate one for our analysis. By comparing the time consumed (Speed) and correct detection percentage (Accuracy) for each model, we finally selected the “SSD_RESNET_50_FPN_COCO” model as it has a faster detection speed (1.27 seconds per images) while is able to present a relatively higher accuracy (81.4%). The detection speed and accuracy of each model is listed in Table 3. The field of object detection is developing fast and new models are released in high frequency in recent years. Although not all latest models are tested, the accuracy of the selected model is higher than 80% and could meet the requirement of this research.

**Table 3:** Comparison of deep convolutional neural network models.

Model Name^a^	Speed (Seconds)	Accuracy (%)
FASTER_RCNN_INCEPTION_RESNET_V2	10.33	86.0
SSD_RESNET_50_FPN_COCO	1.27	81.4
MASK_RCNN_RESNET101_ATROUS_COCO	7.83	76.7
SSD_MOBILENET_V1_FPN_COCO	0.93	74.4
FASTER_RCNN_RESNET50_COCO	1.48	69.8
RFCN_RESNET101_COCO	1.53	69.8
FASTER_RCNN_INCEPTION_V2_COCO	0.97	65.1
SSD_INCEPTION_V2_COCO	0.70	55.8
SSD_MOBILENET_V2_COCO	0.52	51.2
SSDLITE_MOBILENET_V2_COCO	0.45	51.2

^a^ The object detection models could be downloaded from https://github.com/tensorflow/models/tree/master/research/object_detection

For each image processed by the deep convolutional neural network, a bundle of information could be retrieved, including: (1) ‘detection_boxes’, which in composed by four values: *Xa*, *Ya*, *Xc*, *Yc*, representing the horizontal and vertical coordinates of the lower left and upper right corner of the object detection frame in the object detection coordinate system, and each coordinate ranges from 0 to 1; (2) ‘detection_socres’, which ranges from 0 to 1 to represent the degree of confidence of the detection result; (3) ‘detection_classes’ that labels the classification of each object being detected (1 for pedestrian, 3 for vehicle, et cetera); (4) ‘Frame_Number’ as the ID of each image. We then filtered the detection boxes by detection classes, and deleted all detection boxes that were not detected as a pedestrian.

### Geo-locating algorithm

As mentioned above, we have obtained the remote sensing image with coordinates of the study area. In the geo-locating process, the projected coordinate system is marked as the “plan coordinate system”, whose X axial is horizontal and Y axial is vertical, and the positive direction of X and Y axial is from left to right and bottom to top. Then the lower left, lower right, upper right and upper left apexes of the minimum enclosing square of the study area in the image coordinate system were marked as *PA*, *PB*, *PC* and *PD*.

Then we select an image that has been converted from the video and put it in a rectangle coordinate system named “image coordinate system”, whose positive direction of X and Y axial is as same as the plan coordinate system. The coordinate origin of the image coordinate system is the lower left corner of the image, and we stretched the image to make sure that the coordinate of its upper right corner is [1, 1] and marked four apexes (*PA*, *PB*, *PC* and *PD)* of the minimum enclosing square of the study area in the image coordinate system as *A*, *B*, *C* and *D*.

As argued previously, the study area should be divided into analysis grids with the size of around 0.9 meters by 0.9 meters. Since the shape of a small public space would have been transformed when its image is recorded by a photographic device, we cannot directly equally divide the study area in videos into subareas. For example, a foursquare shape space could be transformed to different convex quadrilaterals when the photographic device is installed differently, and there is no way to divide a random convex quadrilateral into given number quadrilaterals with the same shape. Besides, for quadrilaterals that could be equally divided into sub-quadrilaterals, the real size of the sub-quadrilaterals near photographic device would always be greater than those farther away. Therefore, we employed geometric methods to divide a regular shape study area into several subareas with equal size that are close to 0.9 meters by 0.9 meters in the real-world based on perspective principles, regardless of the shape of it in videos and images.

**Fig 2:**
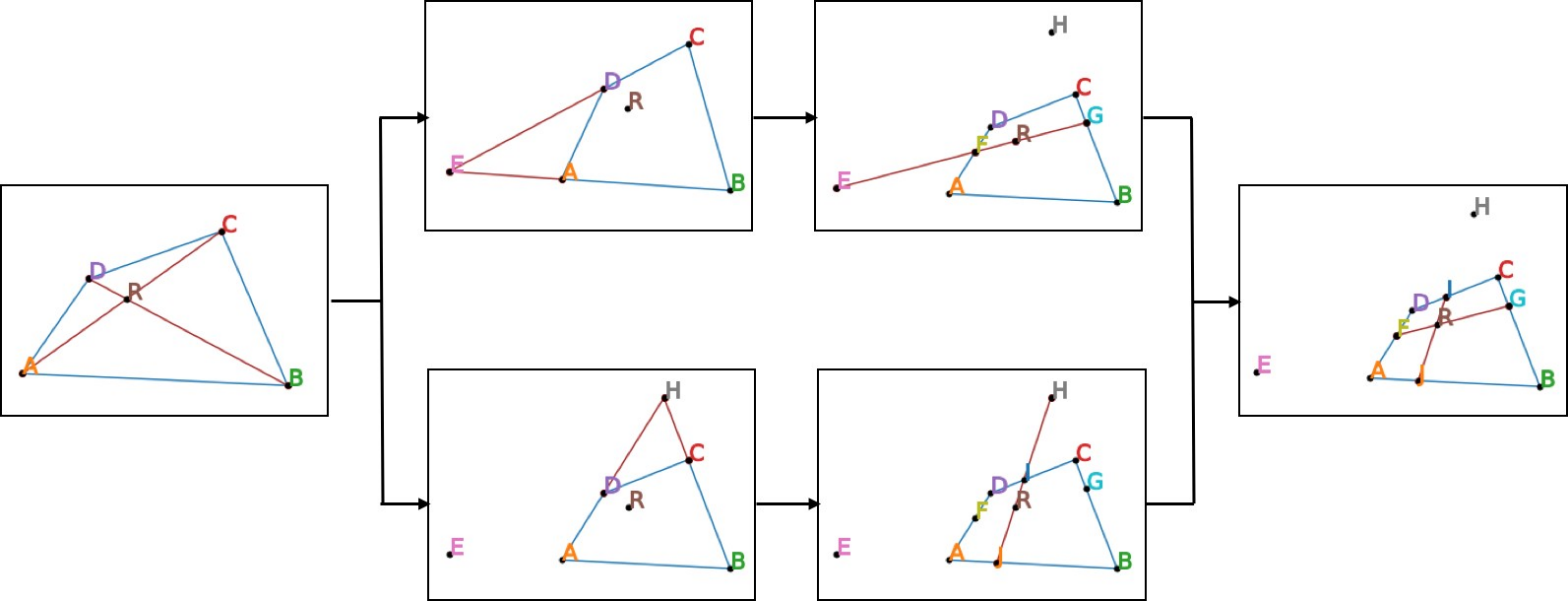
Division of the polygon ABCD into equal size subareas.

The first step is to divide the minimum enclosing square of the study area (see the polygon *ABCD* below) in the image coordinate system into four subareas with equal size in the plan coordinate system. For that purpose, we intersected segment lines *AC* and *BD* to get the intersection point R, extended segment lines *CD* and *BA* to get the intersection point E, and extended segment lines *AD* and *BC* to get the intersection point *H*. If segment lines *AB* and *CD* or *AD* and *BC* are parallel lines, the intersection point *E* or *H* wouldn’t exist. In this circumstance, we could draw a line that parallel to segment line *AB* or *BC* through intersection point *R*, to replace segment line *ER* or *HR*. Then we extended the new segment line *ER* which intersects sides *AD* and *BC* at points *F* and *G*, and extended the new segment line *HR* which intersected sides *CD* and *AB* at points *I* and *J*. Finally, we got four new polygons: *AJRF*, *JBGR*, *RGCI* and *FRID*. Their sizes are all different in images, but those four areas they cover in the real-world space have the same area based on perspective principles (Fig 2).

By repeating the process above, the polygon *ABCD* could be further divided into 16, 64, 256 … *4*^*n*^ subareas, which could be used as analysis grids. The times that the polygon ABCD could be divided (*n*) is calculated as below:

$$n={\left(L/GW\right)}^{0.25}-1,$$ (2)

where *L* is the lengh of the minimum enclosing square of the study area, which could be calculated by the coordinates of *PA* and *PC* in the plan system; *GW* is the length of each analysis grid, which is 0.9 meter in this study. After dividing the minimum enclosing square of the study area *n* times using recursive algorithm, we have obtained *4*^*n*^ analysis grids and the coordinates of their corners in the image coordinate system.

Using the coordinates of the preserved detection boxes’ lower left and upper right corners we obtained in the object detection process, the coordinate of the detection box’s bottom midpoint could be calculated as:

[Xc,(Ya+Yc)/2], (3)

which represents the coordinates of people’s feet in the object detection coordinate system. Since the directions of x axial and y axial are different in the object detection and image coordinate system, the coordinate of the detection box’s bottom midpoint in image coordinate system has to be converted as below:

[(Ya+Yc)/2,1−Xc]. (4)

Then we counted the total number of persons in each analysis grid by matching coordinates of detected objects with the *4*^*n*^ analysis grids’ boundary in the image coordinate system. Next, we used the geometric methods which is mentioned before to divide the minimum enclosing square of the study area in the plan coordinate system into *4*^*n*^ analysis grids, for we have obtained the four apexes’ coordinate (*PA*, *PB*, *PC* and *PD*) of it. Since the apexes *PA*, *PB*, *PC* and *PD* in the plan coordinate system could strictly matched to apexes *A*, *B*, *C* and *D* in the image coordination system, the *4*^*n*^ analysis grids in the image coordination system could also match those in the plan coordinate, which means the count of persons in each analysis grid in the image coordination system also shows the count of persons in each analysis grid in the plan coordination system. Given that the plan coordination system could reflect the geometric information of the real world, we have obtained the total number of persons in the equal-sized analysis grids in the minimum enclosing square of the study area. Then we removed the grids that were out of the boundary of the study area, and finally acquired the usage of a small public space.

Analysis grids are generated from the recursive function aforementioned, the permutation order is not the order of columns and rows in final analysis grids, but shows counterclockwise order in loops. Therefore, it is very hard to understand the result of small public spaces usage by directly reading the number of persons in each analysis grids showing in the final result list, so visualizing the number of persons in each analysis grid is the last but necessary step. We took the number of persons as the color parameters of analysis grids, and eventually the statistical result is displayed by color ramp.

## Validation experiment

**Fig 3:**
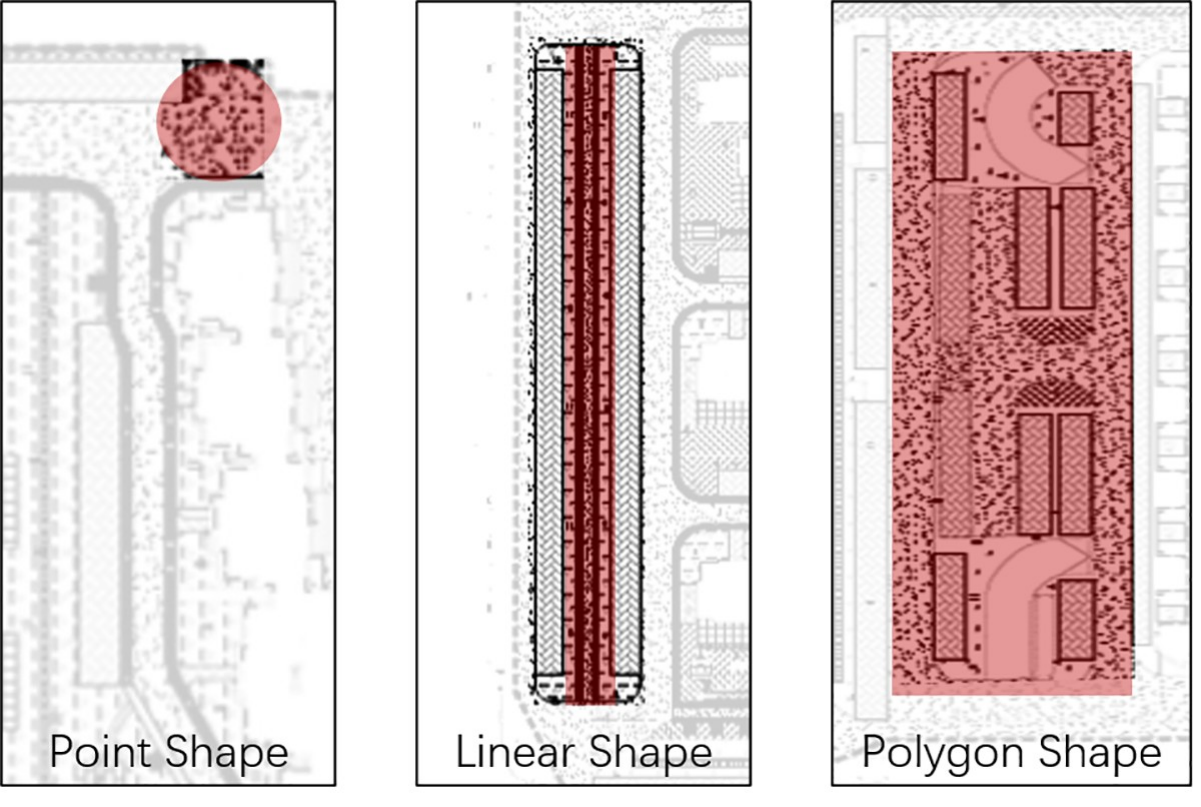
Geometric shapes of small public spaces.

To demonstrate the applicability of our approach, we experimented it in a real-world community. In the case study, we tested our approach in small public spaces of different geometric shapes, including point shape, linear shape and polygon shape (Fig 3). Point shape public space can be found at the entrance or exit of a residential community. Linear shape space is usually a linear space such as road and green corridor. Polygon shape space refers to the small public space with complex activity paths, like garden, playground or public parking lots, which are relative larger in size.

### Experiment design

We have selected a residential community in Tongzhou District, Beijing as the study area. Before conducting the experiment, we have obtained the permission from the property management company for conducting social observation experiments by installing photographic devices. We have also signed an agreement with the property management, stating that all the data collected during the experiment would only be used for research purposes, and the data containing privacy information of the property owners will not be shared with the third party unless agreed upon by the property management company, and the facial information in the images needed to be blurred before it is released.

**Fig 4:**
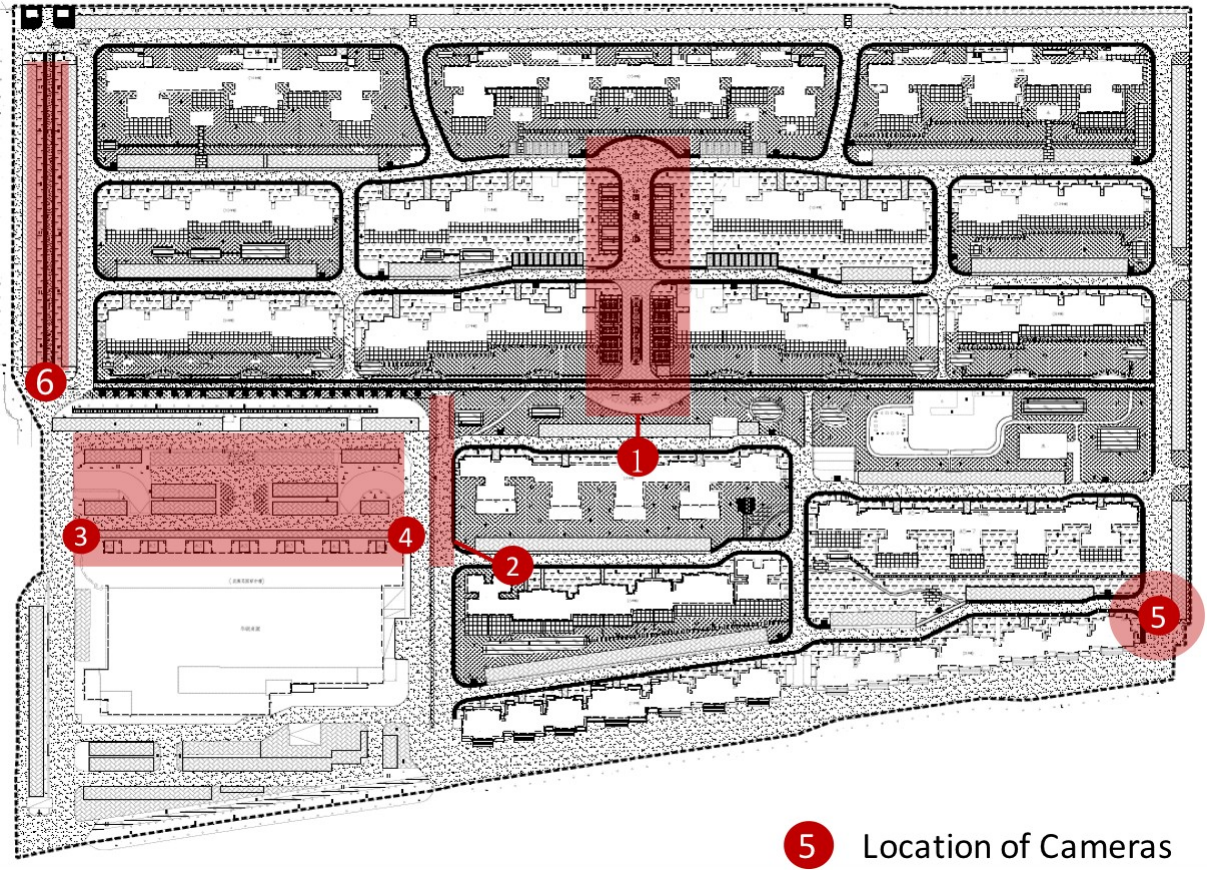
Location of photographic devices.

There are five small public spaces in three different shapes and size in this community: two corridors as small and large linear shape spaces, a garden and a parking lot as small and large polygon shape spaces, and a community entrance as a point shape space (Fig 4). Whether a study area is small or large is defined as follow: if the minimum enclosing square of a study area could be recorded by a photographic devices installed in low position, then it is a small study area, otherwise it is a large study area. Six photographic devices were installed to collect video data of these public spaces simultaneously. For the point shape space, the Camera 5 was installed in low position to collect videos of the community entrance, for the video recorded from low position can cover the entire space with higher resolution. As for the linear shape spaces, Camera 6 was installed in low position and Camera 2 was mounted in high position to collect videos of a small and a large corridor to test whether the installation height and the size of the study area would significantly influence the detection results. For the polygon shape spaces, Camera 1 was installed in high position to collect videos of the garden, while Camera 3 and 4 were installed in low position on both side of the parking lot. Considering the size of the polygon shape space, it is impossible to collect its images by a single camera in low position. Therefore, we tried to collect images of a polygon shape space by installing two cameras in low position on each side of the public parking lot, while mounting another camera at high position in the middle to collect data of the garden. By testing whether people who walked by or stayed in two spaces were all recorded, we could compare the two ways of collecting data of small and large polygon small public spaces. Table 4 shows where each photographic device was installed.

**Table 4:** Installation of photographic devices.

Small public space	Device Name	Installation Position	Geometric Shape	Size of the Study Area
Community Entrance	Camera 5	Low (3.5m)	Point Shape	Small
North Corridor	Camera 6	Low (4m)	Linear Shape	Small
South Corridor	Camera 2	High (8m)	Linear Shape	Large
Parking Lot	Camera 3 and 4	Low (3.5m)	Polygon Shape	Small
Garden	Camera 1	High (7m)	Polygon Shape	Large

After the preparation, 15 minutes of videos were recorded by each camera in a cloudy weekday from 9:30 am to 9:45 am. The record time was chosen to guarantee that each camera could record 30 to 100 people in those small public spaces. The reason to pick a cloudy day is to avoid the influence from shadow effects in a sunny day, which may affect the detection results. After the image conversion with time interval as 0.75 seconds per image, 1200 images were filtered from videos recorded by each camera for further object detection and quantitative analysis.

### Results and analysis

**Fig 5:**
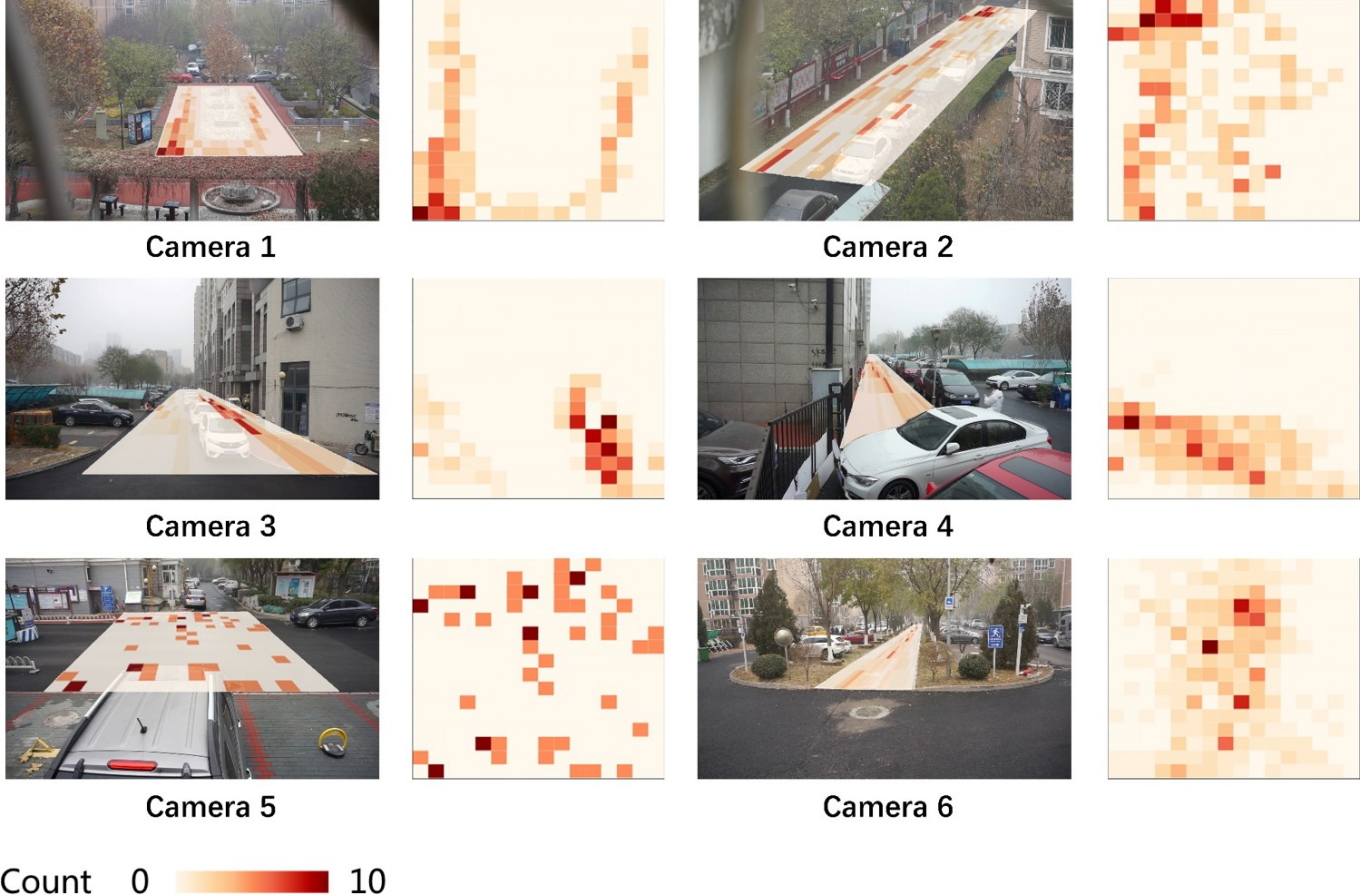
The usage of small public spaces.

In the object detection process, the location and class of each object in images were obtained by using deep convolutional neural network. Six datasets containing the information of people in each public space were generated using the approach we introduced before. In order to geo-locate people in images to the site plan of each public space, we calculated coordinates of each public space’s vertexes, and divided each public space into 256 analysis grids considering their size. Finally, we geo-located and visualized detected objects into analysis grids (Fig 5).

Camera 1 recorded the usage of the garden square in the community. It showed that people usually entered the square from the north entrance and leave the square at the southwest corner. Besides, people tended to walk along the edge of a space rather than along the diagonal line when choosing a moving path.

Camera 2 recorded the usage of the corridor in the middle south, which showed that people usually walked on the edge of a road. Yet when there were vehicles and garbage cans along the edge of the road, people would like to keep a distance from them. The northwest side of the road is an extra entrance, which made it a place with high spatial vitality.

Camera 3 and 4 recorded the usage of the parking lot, and the results indicated that when people were walking on the sidewalk along a building, they would prefer to keep away from the building rather than close to it. People who were walking in parking lots usually moved from northeast to southwest heading to the nearest exit.

Camera 5 recorded how people passed the community entrance. It is difficult to construct a continuous path by using the previous time interval, which reflected that people tended to walk faster when entering or exiting the community gate than other places. The results suggested that pedestrians had to move to the middle of the road from time to time to avoid vehicles from both sides because there is no sidewalk in the area.

Camera 6 recorded the usage of the northwest sidewalk corridor, and the result implied that this corridor was the most popular public space for relaxation and entertainment, since there were many benches and frondent green plants on both sides. Most people entered from the south of the corridor and then moved to the middle of the space while keeping a certain distance from the seats on both sides in avoiding of either getting trapped by them or disturbing people resting on benches.

### Method validation

Using deep convolutional neural network to quantify the usage of small public spaces from videos is a promising methodology compared with experiments utilizing observation methods, because observation methods are heavily limited by the speed and accuracy of marking down the location of people, time of observation and cost for hiring qualified observer. Since our method is based on automatic identification and processing, its time consumption and labor costs are definitely lower than traditional method. So the main goal of method validation is to verify the effectiveness and efficiency of our approach. In the validation process, a manual audit approach is implemented as the comparative experiment. To start with, we selected 1272 images (212 images from each photographic device) containing persons from all images converted from videos recorded in the experiment. Then we observed and counted people of each selected image, and manually marked the number of persons in each analysis grid. After that, we mapped the result on each image and compared it with results we got by using deep convolutional neural network and geometry algorithm. The result of the method validation is listed in Table 5.

**Table 5:** Method validation.

Device Name	Position	Accuracy	Missing Ratio	False Ratio
Camera 1	High	97.2%	0%	2.8%
Camera 2	High	96.8%	0%	3.2%
Camera 3	Low	96.0%	4%	0%
Camera 4	Low	98.1%	1.9%	0%
Camera 5	Low	98.4%	1.6%	0%
Camera 6	Low	97.7%	1.8%	0.5%

For those tested images, our approach delivered a accuracy greater than 95% for all three types of geometric shapes of small urban places, which identified the reliability and accuracy of our approach. In spite of that, there are two issues that need to be noted. The first is that the height of the device may influence the accuracy of object detection result. For videos recorded in high position, the false ratio is higher than those recorded in low position, since most of the pictures that were used to train the object detection model were taken from normal height around 1.5 meters, which is much lower than the height of Camera 1 and 2. This could also explain the differences of false ratio of photographic devices that were installed in both low and high position: for the photographic device installed in high positon, the false ratio of Camera 2 is higher, as it was the highest (8 m); for the photographic device installed in low positon, the false ratio of Camera 3 is the highest, since it was higher than other low position devices. The second issue is that although the videos were not recorded in rush hour, the missing ratio of the videos recorded in low position is higher than those recorded in high position, due to the overlap of people and vehicles in the images. The two issues generate the tradeoffs between high and low position. Since the accuracy of object detection model could be increased by training the model with videos recorded from high position and the missing ratio would be even higher in dense situation if the photographic device is installed in low position, it is recommended to install the photographic devices in high position.

## Conclusions and discussion

In this paper, we proposed a novel approach of quantifying the usage of small public spaces using deep convolutional neural network and validated our approach by conducting a real-world experiment with empirical data we collected. In detail, we utilized a deep convolutional neural network to automatically detect people in videos collected before, and then, proposed a geo-locating algorithm to convert their locations from image-based positions to real-world projected coordinates by grid division and to count people in each grid automatically. Eventually, the cumulative number of persons per grid can reflect the usage of a space on a fine scale. To validate the accuracy and applicability of our approach, we selected six experimental sites and compared results generated from our new approach with those from a manual audit. The experimental results proved the reliability and robustness of our method.

### Academic contributions

Compared with previous studies, our new approach has four merits and features. First, video data has high accuracy and limited system error, for it could record all the physical environment and human activities in small public spaces and can be checked and verified in both data collecting and result process. Second, spatial elements in small public spaces and their possible influences on people’s behaviors could be directly observed from the results, or by the way of geo-locating the spatial elements. Third, the time granularity of this method is high and the time interval could be changed according to the research purpose. Last but not least, the spatial resolution of this approach can be also high by dividing the space into finer scale analysis grids. Therefore, this method can be used for more detailed studies of a specific space and has low information redundancy.

### Potential applications

This study is an initial attempt to automatically map the usage of small public spaces. After validation, this approach shows its feasibility for fine-scale spatial-temporal behavior research, suggesting its potential applications in evaluating the design of public spaces and shaping urban designs to the dimensions of humanization and individualization. Considering the accessibility of data collection, it can be used to analyze the small public spaces in different areas and cities.

Specifically, the application prospects of this approach can be mainly embodied in three dimensions. First, it could be applied in representing the usage of public space and quantitatively measuring the design implementation, so as to evaluate the quality of the design. Second, designers could discover main elements impacting on people’s behaviors via the fine-scale spatial observation, so that they can optimize their design to match the growing demand for human-oriented space. Moreover, as the approach can help explore the relationship between crowd activities and environmental elements from the perspective of environmental behavior, it is conducive to expanding the fundamental theory of small public space studies.

### Potential limitations and future research

While admitting the merits of our method, there are still several avenues requiring further investigation in the close future. From the perspective of method’s algorithms, some optimization algorithms need to be applied to solve the limitations of the existing method. On the one hand, in the public space division process, the number of analysis grids has to be 4^*n*^, which limits the flexibility of this method. For a small public space with a very high or very low width-length ratio, we have to get coordinates of a square space that covers the public space from videos, to guarantee the size of each analysis grid similar to a 0.9 meters x 0.9 meters’ square. On the other hand, if the outer square space is too large, we may not be able to get its precise coordinates from videos. In the future study, we would develop a new algorithm to divide small public spaces by any given numbers to make sure that the size each analysis grid is precise 0.9 meters x 0.9 meters. From the perspective of method application, this research only chooses public spaces in residential areas as experimental sites to validate our method. More types of space, such as squares in campus, business districts and railway stations etc. need to be further studied.

## Supporting information

S1 File Minimal anonymized data set. (RAR)
